# IFNAR1 gene mutation may contribute to developmental stuttering in the Chinese population

**DOI:** 10.1186/s41065-021-00211-y

**Published:** 2021-11-18

**Authors:** Yimin Sun, Yong Gao, Yuxi Zhou, Yulong Zhou, Ying Zhang, Dong Wang, Li-Hai Tan

**Affiliations:** 1 State Key Laboratory of Membrane Biology, School of Medicine, Tsinghua University, Beijing, 100084 China; 2 Medical Systems Biology Research Center, School of Medicine, Tsinghua University, Beijing, 100084 China; 3 Beijing CapitalBio Technology Co., Ltd., Beijing, 101111 China; 4 Guangdong-Hongkong-Macau Institute of CNS Regeneration and Ministry of Education CNS Regeneration Collaborative Joint Laboratory, Jinan University, Guangzhou, 510400 China; 5 Center for Language and Brain, Shenzhen Institute of Neuroscience, Shenzhen, 518060 China; 6 State Key Laboratory of Southwestern Chinese Medicine Resources, School of Basic Medical Sciences, Chengdu University of Traditional Chinese Medicine, Chengdu, 611137 China

**Keywords:** IFNAR1, Developmental stuttering, Whole-exome sequencing (WES), Persistent developmental stuttering (PDS)

## Abstract

**Background:**

Developmental stuttering is the most common form of stuttering without apparent neurogenic or psychogenic impairment. Recently, whole-exome sequencing (WES) has been suggested to be a promising approach to study Mendelian disorders.

**Methods:**

Here, we describe an application of WES to identify a gene potentially responsible for persistent developmental stuttering (PDS) by sequencing DNA samples from 10 independent PDS families and 11 sporadic cases. Sanger sequencing was performed for verification with samples obtained from 73 additional patients with sporadic cases.

**Results:**

We first searched for cosegregating variants/candidate genes in a Chinese family (Family 0) by sequencing DNA obtained from 3 affected members and 3 controls. Next, we sequenced DNA samples obtained from 9 additional Chinese families (Families 1-9) with stuttering to verify the identified candidate genes. Intriguingly, we found that two missense variants (Leu552Pro and Lys428Gln) of interferon-alpha/beta receptor 1 (IFNAR1) cosegregated with stuttering in three independent families (Families 0, 5 and 9). Moreover, we found two additional mutations (Gly301Glu and Pro335del) in the IFNAR1 gene in 4 patients with sporadic cases by using WES or Sanger sequencing. Further receptor mutagenesis and cell signaling studies revealed that these IFNAR1 variants may impair the activity of type I IFN signaling.

**Conclusion:**

Our data indicate that IFNAR1 might be a potential pathogenic gene of PDS in the Chinese population.

**Supplementary Information:**

The online version contains supplementary material available at 10.1186/s41065-021-00211-y.

## Introduction

Stuttering is speech dysfluency with typical symptoms include involuntary interruption, repetition or prolongation of words or syllables during verbal communications [1]. There are three subtypes of stuttering: developmental stuttering, neurogenic stuttering and psychogenic stuttering [2]. The most common form of stuttering is developmental stuttering, which accounts for more than 80% of total stuttering cases. It is an isolated form of stuttering without apparent neurogenic or psychogenic impairment. The prevalence of developmental stuttering among preschool children is approximately 5%. Practically, more than 75% of children with developmental stuttering spontaneously stop stuttering before adulthood. However, a portion of developmental stuttering can persist into adulthood as persistent developmental stuttering (PDS). It was reported that 1% of the global population is affected by PDS [3]. Although the etiologies and pathologies of PDS remain elusive, there has been increasing evidence indicating considerable heritability but only moderate environmental factors underlying the incidence of PDS [4].

Previous linkage studies led to the proposal four susceptibility loci for PDS that are archived in the OMIM database as STUT1 (Stuttering, Familial Persistent 1) to STUT4. The first PDS locus discovered, STUT1, is located on chromosome 18 and was identified in a genome-wide linkage scan of families in North America and Europe. The transmission pattern of this PDS locus was consistent with autosomal dominant inheritance [5]. Then, through studies investigating inbred families in Pakistan, cosegregated genetic loci or variants underlying PDS were more directly identified. Indeed, a number of novel PDS loci were identified in Pakistani families, including STUT2 on chromosome 12 [6], STUT3 on chromosome 3q [7], and STUT4 on chromosome 16q [8]. Notably, the STUT2 locus was further refined in a large, highly inbred Pakistani family. Mutations identified in genes, including N-acetylglucosamine-1-phosphate transferase subunits alpha and beta (GNPTAB), N-acetylglucosamine-1-phosphate transferase subunit gamma (GNPTG) and N-acetylglucosamine-1-phosphodiester alpha-N-acetylglucosaminidase (NAGPA), suggested a pathogenic role of the lysosomal enzyme-targeting pathway in PDS [9]. In addition, a number of PDS studies have been performed with outbred families in which genetic etiologies could be more complex. A linkage study in a large African family did not lead to the identification of any single locus showing significant linkage to PDS [10]. When the large pedigree was divided into five subpedigrees, however, multiple novel loci emerged within each subpedigree. More recently, it was reported that rare coding variants of the adaptor related protein complex 4 subunit epsilon 1 (AP4E1) gene cosegregate with PDS in one of the aforementioned subpedigrees of this African family, and it was proposed to possibly contribute to deficits in intracellular trafficking in PDS [11]. In addition to family studies, a number of studies of sporadic and unrelated stuttering cases have been performed, and potential pathogenic genes, including solute carrier family 6 member 3 (SLC6A3), forkhead box P2 (FOXP2) and contactin associated protein 2 (CNTNAP2) [12–14], have been proposed with nominal evidence.

To date, these proposed genes can only explain a limited fraction of the heritability underlying PDS. The genetic etiologies of PDS are believed to be polygenic given substantially different linguistic and genetic backgrounds among global populations. To our knowledge, existing genetic studies of stuttering have mainly been performed among English-speaking populations such as Caucasians and Africans. Given the complex and varied language processing differences between English and Chinese speech, PDS in Chinese stutterers might be different than it in English speakers. Therefore, studies in Chinese families might provide additional explanations for the high heritability underlying PDS.

In the present study, we performed whole-exome sequencing (WES) analysis and/or Sanger sequencing in selected affected members in 10 families and 84 patients with sporadic cases to identify potential pathological genes associated with PDS. Our preliminary results suggest that the interferon-alpha/beta receptor 1 (IFNAR1) gene might be a novel PDS-associated gene in Chinese stutterers.

## Methods

### Subjects

Family 0 was recruited from Shandong Province in China (Supplementary Table S1; 7 participants in all; 6 samples were subjected to WES). Families 1-9 were recruited from Henan Province in China (Supplementary Table S1; 38 participants in all; 33 samples were subjected to WES). All family members were native Chinese speakers. A self-reported history of stuttering and symptoms was obtained through face-to-face interviews with each participant. Then, stuttering was diagnosed according to the Stuttering Severity Instrument-III (SSI-3). All of the subjects reported no neurological or language problems except for stuttering. Moreover, 84 unrelated sporadically affected individuals were recruited from several stuttering correction centers, and the population-matched control individuals consisted of age-matched and sex-matched persons with normal speech from the same regions of China (Supplementary Table S1; 84 participants; 11 samples were subjected to WES). This study was approved by the institutional review board of Tsinghua University School of Medicine. All participants signed written informed consent forms and provided saliva samples.

### Whole-exome sequencing

Oragene^TM^ DNA self-collection kits OG-500 (Ottawa, Canada) were used to collect saliva from each participant. Genomic DNA was extracted according to the manufacturer’s instructions. NanoDrop 1000 (Waltham, U. S) and Qubit 2.0 (Grand Island, U. S) were used to determine DNA concentrations. For library construction, purified DNA samples were randomly fragmented into 150–200 bp lengths. The fragments were ligated with an adapter and amplified by ligation-mediated PCR. Roche NimbleGen SeqCap EZ Exome kits (Madison, U. S) was used for exon capture. Illumina HiSeq 2500 (San Diego, U. S) was used for high-throughput sequencing. Raw sequencing reads were mapped to a reference genome (GRCh37/hg19). Single-nucleotide variations (SNVs) and insertions/deletions (indels) in each sample were annotated with chromosome position, alleles, dbSNP ID, minor allele frequency (MAF), gene, variant types, sequencing depth and quality. WES and bioinformatics annotation were performed at CapitalBio Corporation (Beijing, China). In addition, the WES results were compared with a group of 202 unrelated healthy controls for which WES data were available. To identify potentially pathogenic variants, we removed (1) synonymous variants or intronic variants and (2) variants with a frequency greater than 0.01 in the 1,000 Genomes Browser.

### Sanger sequencing

For stuttering families (Families 0, 5 and 9), Sanger sequencing was performed on the detected mutation sites of the IFNAR1 gene to exclude false positives and to verify the presence of cosegregation in the indicated samples (Supplementary Table S1). DNA fragments containing candidate variants were amplified by polymerase chain reaction (PCR). For 73 sporadic cases, all exons of the IFNAR1 gene were sequenced to identify potential variants of the IFNAR1 gene. The quality of the PCR products was examined by 1.5% agarose gel electrophoresis. Next, a 3730xl DNA Analyzer (Grand Island, U. S) was used to perform Sanger sequencing. Laboratory experiments were performed at BGI (Wuhan, China) and TSINGKE Biological Technology (Beijing, China). The results of the Sanger sequencing were evaluated by Chromas (v.2.4.4).

### In vitro study of the effects of IFNAR1 variants found in stuttering

To explore the functional effect of IFNAR1 variants, HEK-293 cells were seeded in 24-well plates (5 × 10^4^/ml/well) 24 h prior to cotransfection of a plasmid DNA pGV208-hIFNAR1-WT (0.5 μg) or pGV208-hIFNAR1-MUT vector (0.5 μg; e.g., pGV208-hIFNAR1-G301E, pGV208-hIFNAR1-K428Q, pGV208-hIFNAR1-L552P, or pGV208-hIFNAR1-del335) with an IFN-stimulated response element (ISRE) reporter (0.5 μg) and the pTK-Renilla Luc control vector (0.02 μg) using X-tremeGENE (Roche Diagnostics, Basel, Switzerland). For the indicated experiments, forty-eight hours after transfection, the cells were stimulated with 100 IU/ml human IFN-β for 12 h. After treatment, the transfected cells were lysed in 100 μl of passive lysis buffer (Promega Corp., Madison, WI, US), and both firefly and Renilla luciferase activity levels were assessed using a dual-luciferase reporter assay system according to the manufacturer’s instructions (Promega). To determine the activity of the ISRE reporter, the lysates assayed for firefly luciferase activity were normalized against Renilla luciferase activity.

## Results

### Identification of candidate variants in IFNAR1

**Fig. 1:**
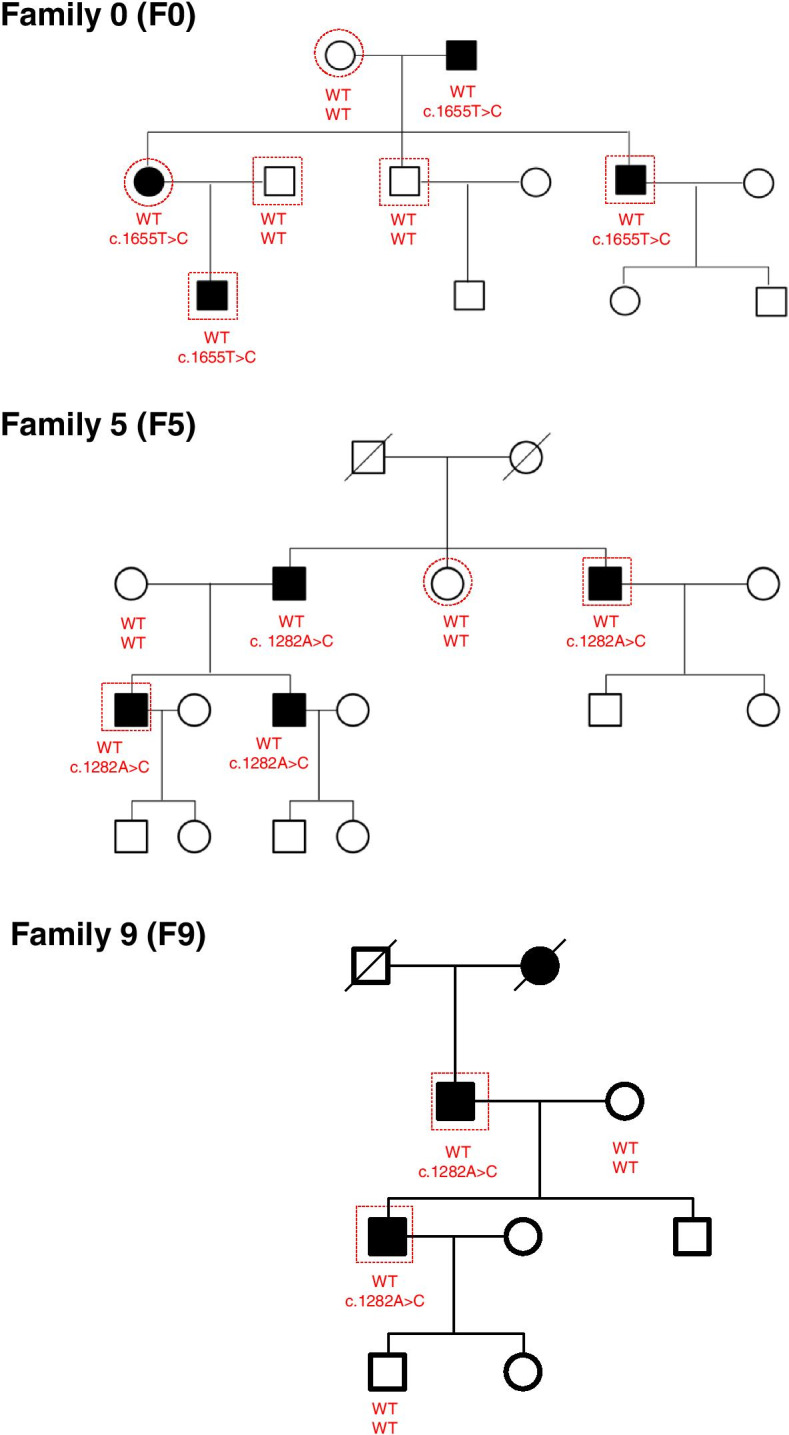
Cosegregation of the chromosome containing the c.1282A>C and c.1655T>C variations of IFNAR1 in Chinese families 0, 5 and 9. Samples collected for WES analysis was indicated as red dashed lines

To identify candidate genes harboring novel pathogenic variants associated with PDS in Chinese stutterers, we first performed WES analysis on samples obtained from 6 members from a Chinese family (Family 0, indicated as dashed lines), including three affected individuals and three unaffected individuals (Fig. 1). No other speech problems except stuttering were observed in these family members during face-to-face interviews. The WES analysis revealed 28 cosegregating SNVs (minor allele frequency [MAF] < 0.01) corresponding to 24 candidate genes (including IFNAR1 and olfactory receptor family 4 subfamily C member 3 [OR4C3]) in Family 0 (Supplementary Table S2).

Next, to investigate whether these variants are associated with stuttering, we analyzed the WES data obtained for members of 9 other Chinese families (Families 1-9) and 11 sporadic cases. The indicated samples were selected and subjected to WES analysis (Supplementary Fig. S1 and Supplementary Table S1). Interestingly, after assessing the MAF (MAF < 0.01) and cosegregation, we observed an IFNAR1 variant (rs563741878) in Families 5 and 9 (Fig. 1), and another variant (rs375386475) in 1 sporadic case (Fig. 1). Our data suggest that IFNAR1 might be associated with stuttering in three families (Families 0, 5 and 9) and sporadic cases, as summarized in Table 1, Fig. 1 and Supplementary Table S3.

**Table 1:** INFAR1 mutations identified in individuals with stuttering

Nucleotide mutation	Amino acid mutation	SNP ID	Family		Individuals	Function
			Case (*N*=66)	Control (*N*=50)	Case (*N*=86)	
1282A>C	Lys428Gln	rs563741878	[F5] **4** [F9] **2**	[F5] 0 [F9] 0	0	Benign (PolyPhen)
1655T>C	Leu552Pro	rs762410025	[F0] **3**	[F0] 0	0	Delerious (SIFT)
902G>A	Gly301Glu	rs375386475	0	0	**1**	probably damaging (PolyPhen)
1002_1004 delTCC	Pro335del	rs72552343	0	0	**3**	Delirious (PROVEAN)

### Validation of the candidate variants in IFNAR1

To validate the candidate variants of the IFNAR1 gene, Sanger sequencing was performed in samples obtained from extended family members of Families 0, 5 and 9, depending on sample availability (Supplementary Table S1). In Family 0, the missense variant of NM_000629.2:c.1655T>C was validated in four members (I:2, II:1, II:5, and III:1) with PDS and absent in three control members (I:1, II:2, and II:3). This variant harbors a substitution in which thymine is replaced with cytosine, which results in an amino acid substitution of leucine with proline (Table 1 and Fig. 1).

In Family 5, the missense variant of NM_000629.2:c.1282A>C was validated in four members (II:2, II:4, III:1, and III:3) with PDS and absent in two members (II:1 and II:3) without PDS. In Family 9, the same missense variant was validated in two members with PDS (II:1 and III:1) and absent in two controls (II:2 and IV:1). This variant harbors a substitution in which adenine is replaced with cytosine, which results in an amino acid substitution of lysine with glutamine (Table 1 and Fig. 1).

Similarly, Sanger sequencing was performed to validate the IFNAR1 variant (NM_000629.2: c.902G>A) in the sporadic case originally identified by WES analysis (Table 1 and Supplementary Fig. S2). Then, we also performed Sanger sequencing analysis of all the exons in the IFNAR1 gene in the samples obtained from other 73 sporadic cases. Interestingly, we identified an additional mutation (p.Pro335del) in 3 different patients (Table 1 and Supplementary Fig. S3).

**Fig. 2:**
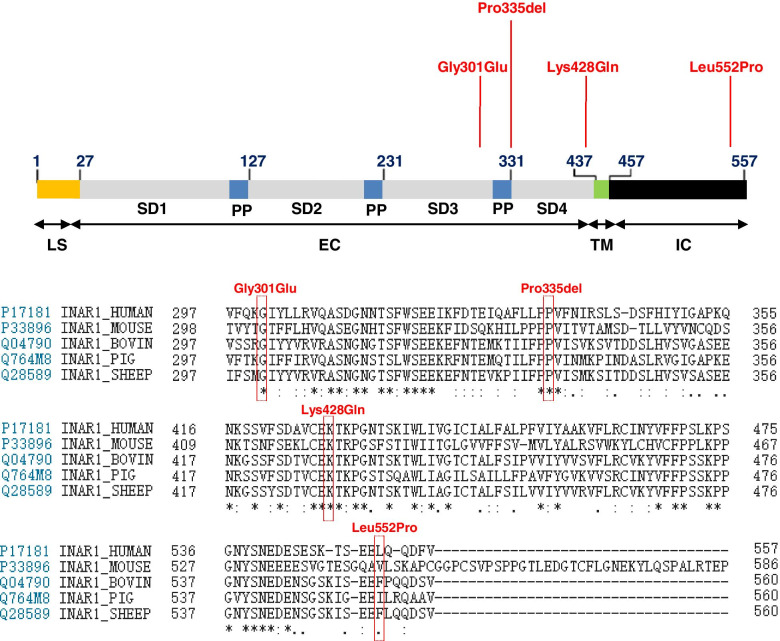
Schematic diagram of the mutations of human IFNAR1 identified in individuals with stuttering. **A** The human IFNAR1 protein is 557 amino acids long and consists of an N-terminal extracellular cytokine-binding (EC) domain (436 aa), a hydrophobic transmembrane (TM) domain (21 aa), and a C-terminal intracytoplasmic (IC) domain (100 aa). **B** The protein sequences of IFNAR1 in different organisms were aligned with the Clustal Omega program in the UniProt database. Its predicted orthologs show the conservation of the Gly301Glu, Pro335 and Lys428 residues. An * (asterisk) indicates positions that have a single, fully conserved residue. **A** (colon) indicates conservation between groups with highly similar properties — scoring > 0.5 in the Gonnet PAM 250 matrix. **A** (period) indicates conservation between groups with weakly similar properties — scoring =< 0.5 in the Gonnet PAM 250 matrix

Collectively, we identified two missense variants of IFNAR1 that cosegregate with PDS and two additional variants in 4 of the 84 sporadic cases (Figs. 1 and 2). Hence, these four variants of IFNAR1 can be considered candidate variants of PDS for use in subsequent functional studies.

### In vitro functional study of the IFNAR1 variants

IFNAR1 encodes interferon-α/β receptor alpha chain, a low-affinity subunit of the interferon-α/β receptor (IFNAR). The rare variants identified in the stuttering subjects mapped to the extracellular and cytoplasmic topological domains (Fig. 2). Moreover, a protein sequence comparison of IFNAR1 with its orthologs across different species revealed the conservation of the Gly301Glu, Pro335 and Lys428 residues (Fig. 2).

**Fig. 3:**
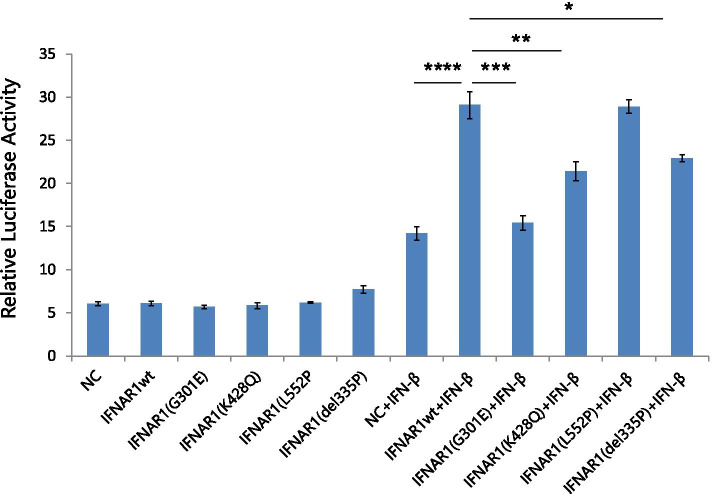
In vitro cell signaling experiments of IFNAR1 variants using a dual-luciferase reporter system. HEK-293 cells were seeded in 24-well plates and then cotransfected with a plasmid DNA pGV208-hIFNAR1-WT or pGV208-hIFNAR1-MUT vector (e.g., pGV208-hIFNAR1-G301E, pGV208-hIFNAR1-K428Q, pGV208-hIFNAR1-L552P, or pGV208-hIFNAR1-del335) together with IFN-stimulated response element (ISRE) reporter and the pTK-Renilla Luc control vector. For the indicated groups, cells were incubated with IFN-β (100 IU/ml) for 12 h. After treatment, the cell lysates were examined to determine the ISRE reporter activity by using the dual-luciferase reporter assay system. *, *P* < 0.05; **, *P* < 0.01; ***, *P* < 0.001

To better understand the biochemical effects of these variants, we constructed vectors that express wild-type and variant interferon-α/β receptor alpha chain proteins. The IFN-stimulated response element (ISRE) luciferase reporter system was applied to determine the relative activity of the IFN signaling pathway with or without IFN-β stimulation. As demonstrated in Fig. 3, when the IFNAR1 vector was mutated, the basal luciferase activity was similar to that of the wild-type vector without IFN-β treatment. After stimulation with IFN-β, the expression levels of the reporter gene in all transfected cells were significantly increased. However, the increase in G301E, E428Q, and P335del vector expression was significantly lower than that of the wild-type vector. However, the increase in the level of the L552P reporter was similar to that of the wild-type vector after IFN-β treatment (Fig. 3). These results indicated that these rare coding variants, except L552P, can impair the response of IFN signal transduction in response to IFN-β treatment.

## Discussion

As a common speech disorder, stuttering is a complex trait with high heritability (often exceeding 80%) and few shared environmental effects in early childhood [15]. However, generally, stuttering does not display a clear Mendelian inheritance pattern, and few genuine monogenic causes for stuttering have been found. To date, GNPTAB, GNPTG, NAGPA and AP4E1 have been associated with stuttering. These four genes encode the lysosomal enzyme targeting pathway, including lysosomal metabolism and endosomal and/or lysosomal trafficking. The estimated contribution of these four genes to stuttering might be close to 20% [16]. Moreover, substantial studies have shown that Chinese and English language processing involve different neural networks and distinct brain area. Specifically, left middle frontal gyrus (LMFG, BA9) might play a crucial role in orthography-to-phonography and orthography-to-semantics conversion during Chinese reading [17–20]. Therefore, the vast majority of genetic causes of stuttering have yet to be identified.

In the present investigation, we identified two heterozygous missense variants of IFNAR1 (p.Leu552Pro and p.Lys428Gln) that cosegregated in three Chinese stuttering families. Further analysis revealed two additional mutations (p.Gly301Glu and p.Pro335del) in 4 of the 84 samples obtained from patients with sporadic PDS. More intriguingly, further cell signaling studies revealed that 3 of these 4 rare variants in IFNAR1 may impair the activity of type I IFN signaling. IFNAR is a heteromeric cell surface receptor composed of two subunits, interferon-α/β receptor alpha chain (encoded by IFNAR1) and interferon-α/β receptor beta chain (encoded by IFNAR2). By binding type I interferon (IFN) cytokines (such as interferons-α, -β, -ε, -κ, -ω, and -ζ), type I IFNs initiate signaling through IFNAR to activate the Janus kinase (JAK)-signal transducer and activator of transcription (STAT) signaling pathway, along with the mitogen-activated protein kinase (MAPK), Phosphoinositide 3-kinases (PI3K), and AKT serine/threonine kinase 1 (Akt) signaling pathways. These signaling pathways facilitate the coordination of multiple biological processes in pathogen infection and multiple autoimmune diseases (e.g., systemic lupus erythematosus, Sjogren's syndrome, systemic sclerosis, rheumatoid arthritis, and myositis) via a downstream transcriptional network [21, 22]. The implicated cellular effects may include apoptosis, cell cycle arrest, and immune modulation [23]. Some studies have shown that pathogen infection is the major nongenetic contribution to stuttering [24] and implied that the immune response may play a role in the occurrence and development of stuttering.

Recently, an in vivo study demonstrated that mice lacking IFN-β/IFNAR signaling exhibited motor and cognitive learning impairments, which were accompanied by defects in neuronal autophagy and Lewy body accumulation [25]. Specifically, the cortical neurons of Ifnb–/– mice showed an increase in autophagosomes and very few autolysosomes compared to WT mice, suggesting blocked autophagic flux [25]. Additionally, another animal study indicated that the type I interferon response is involved in the development of neuronopathic Gaucher disease and possibly in other lysosomal storage diseases in which simple glycosphingolipids accumulate. Here, we showed that multiple IFNAR1 variants significantly impaired IFN-β-induced reporter activity, suggesting that abnormal IFN-β/IFNAR signaling may contribute to a cascade of neurodegenerative events in stuttering cases. Consistent with this notion, our data support previous investigations showing that mutations in the lysosomal enzyme-targeting pathway (including GNPTAB, GNPTG, NAGPA and AP4E1 genes) are associated with persistent stuttering [9, 11, 26, 27]. These findings support the notion that lysosomal dysfunction may lead to stuttering pathogenesis. However, whether these variants in IFNAR1 ultimately impact autophagy or lysosome degradation in vivo remains to be determined in the future by using in vivo models.

## Conclusion

We found that rare coding variants of IFNAR1 occurred in 3 independent Chinese stuttering families and 4 of the 84 Chinese patients with sporadic stuttering (4.8%). Further functional studies demonstrated that these variants may impair the IFN-β/IFNAR signaling cascade. In view of the biological significance of type I IFN signaling in neurodegenerative events such as abnormal autophagy and lysosome dysfunction, we propose that our investigation establishes an association between the IFNAR1 gene and developmental stuttering. Further in vivo experiments will be helpful in clarifying the underlying mechanism.

## Supplementary Information

**Additional file 1: Figure S1.** Samples from stuttering families (1-4 and 6-8) were selected for exome sequencing as indicated by red dashed lines.

**Additional file 2: Figure S2.** Confirmation of the presence of the IFNAR1 902G>A point mutation by Sanger sequencing.

**Additional file 3: Figure S3.** Confirmation of the presence of the IFNAR1 1002_1004 delTCC mutation by Sanger sequencing.

**Additional file 4: Supplementary Table S1.** Samples evaluated in the present study.

**Additional file 5: Supplementary Table S2.** The WES analysis revealed 28 cosegregating SNVs corresponding to 24 candidate genes in Family 0.

**Additional file 6: Supplementary Table S3.** Functional annotation of IFNAR1 mutation using in silico prediction tools.

## Data Availability

All data analyzed in our study are available upon reasonable request.

## Abbreviations

PDS: Persistent developmental stuttering
IFNAR1: interferon-alpha/beta receptor 1
STUT1: Stuttering, Familial Persistent 1
GNPTAB: N-acetylglucosamine-1-phosphate transferase subunits alpha and beta
GNPTG: N-acetylglucosamine-1-phosphate transferase subunit gamma
NAGPA: N-acetylglucosamine-1-phosphodiester alpha-N-acetylglucosaminidase
AP4E1: Adaptor related protein complex 4 subunit epsilon 1
SLC6A3: Solute carrier family 6 member 3
FOXP2: Forkhead box P2
CNTNAP2: Contactin associated protein 2
IFN: Interferon
JAK-STAT: Janus kinase-signal transducer and activator of transcription
MAPK: mitogen-activated protein kinase
PI3K: Phosphoinositide 3-kinases
Akt: AKT serine/threonine kinase 1

## References

[1] Prasse JE, Kikano GE (2008). Stuttering: an overview. Am Fam Physician.

[2] Seery CH (2005). Differential diagnosis of stuttering for forensic purposes. Am J Speech Lang Pathol.

[3] Yairi E, Ambrose N (2013). Epidemiology of stuttering: 21st century advances. J Fluen Disord.

[4] Rautakoski P, Hannus T, Simberg S, Sandnabba NK, Santtila P (2012). Genetic and environmental effects on stuttering: a twin study from Finland. J Fluen Disord.

[5] Shugart YY, Mundorff J, Kilshaw J, Doheny K, Doan B, Wanyee J (2004). Results of a genome-wide linkage scan for stuttering. Am J Med Genet A.

[6] Riaz N, Steinberg S, Ahmad J, Pluzhnikov A, Riazuddin S, Cox NJ (2005). Genomewide significant linkage to stuttering on chromosome 12. Am J Hum Genet.

[7] Raza MH, Riazuddin S, Drayna D (2010). Identification of an autosomal recessive stuttering locus on chromosome 3q13.2-3q13.33. Hum Genet.

[8] Raza MH, Amjad R, Riazuddin S, Drayna D (2012). Studies in a consanguineous family reveal a novel locus for stuttering on chromosome 16q. Hum Genet.

[9] Kang C, Riazuddin S, Mundorff J, Krasnewich D, Friedman P, Mullikin JC (2010). Mutations in the lysosomal enzyme-targeting pathway and persistent stuttering. N Engl J Med.

[10] Raza MH, Gertz EM, Mundorff J, Lukong J, Kuster J, Schaffer AA (2013). Linkage analysis of a large African family segregating stuttering suggests polygenic inheritance. Hum Genet.

[11] Raza MH, Mattera R, Morell R, Sainz E, Rahn R, Gutierrez J (2015). Association between rare variants in AP4E1, a component of intracellular trafficking, and persistent stuttering. Am J Hum Genet.

[12] Lan J, Song M, Pan C, Zhuang G, Wang Y, Ma W (2009). Association between dopaminergic genes (SLC6A3 and DRD2) and stuttering among Han Chinese. J Hum Genet.

[13] Han TU, Park J, Domingues CF, Moretti-Ferreira D, Paris E, Sainz E (2014). A study of the role of the FOXP2 and CNTNAP2 genes in persistent developmental stuttering. Neurobiol Dis.

[14] Petrin AL, Giacheti CM, Maximino LP, Abramides DV, Zanchetta S, Rossi NF (2010). Identification of a microdeletion at the 7q33-q35 disrupting the CNTNAP2 gene in a Brazilian stuttering case. Am J Med Genet A.

[15] Dworzynski K, Remington A, Rijsdijk F, Howell P, Plomin R (2007). Genetic etiology in cases of recovered and persistent stuttering in an unselected, longitudinal sample of young twins. Am J Speech Lang Pathol.

[16] Frigerio-Domingues C, Drayna D (2017). Genetic contributions to stuttering: the current evidence. Mol Genet Genomic Med.

[17] Ge J, Peng G, Lyu B, Wang Y, Zhuo Y, Niu Z (2015). Cross-language differences in the brain network subserving intelligible speech. Proc Natl Acad Sci U S A.

[18] Siok WT, Niu Z, Jin Z, Perfetti CA, Tan LH (2008). A structural-functional basis for dyslexia in the cortex of Chinese readers. Proc Natl Acad Sci U S A.

[19] Tang Y, Zhang W, Chen K, Feng S, Ji Y, Shen J (2006). Arithmetic processing in the brain shaped by cultures. Proc Natl Acad Sci U S A.

[20] Wu J, Lu J, Zhang H, Zhang J, Yao C, Zhuang D (2015). Direct evidence from intraoperative electrocortical stimulation indicates shared and distinct speech production center between Chinese and English languages. Hum Brain Mapp.

[21] de Weerd NA, Nguyen T (2012). The interferons and their receptors--distribution and regulation. Immunol Cell Biol.

[22] Ivashkiv LB, Donlin LT (2014). Regulation of type I interferon responses. Nat Rev Immunol.

[23] Schreiber G, Piehler J (2015). The molecular basis for functional plasticity in type I interferon signaling. Trends Immunol.

[24] Alm PA (2020). Streptococcal infection as a major historical cause of stuttering: data, mechanisms, and current importance. Front Hum Neurosci.

[25] Ejlerskov P, Hultberg JG, Wang J, Carlsson R, Ambjorn M, Kuss M (2015). Lack of neuronal IFN-beta-IFNAR causes lewy body- and parkinson's disease-like dementia. Cell..

[26] Kazemi N, Estiar MA, Fazilaty H, Sakhinia E (2018). Variants in GNPTAB, GNPTG and NAGPA genes are associated with stutterers. Gene..

[27] Benito-Aragon C, Gonzalez-Sarmiento R, Liddell T, Diez I, d'Oleire Uquillas F, Ortiz-Teran L (2020). Neurofilament-lysosomal genetic intersections in the cortical network of stuttering. Prog Neurobiol.

