# Supplementary figures and images for: IFNAR1 gene mutation may contribute to developmental stuttering in the Chinese population
Source: Hereditas. 2021 Nov 18;158:46. doi: 10.1186/s41065-021-00211-y (PMC8600687; doi:10.1186/s41065-021-00211-y)

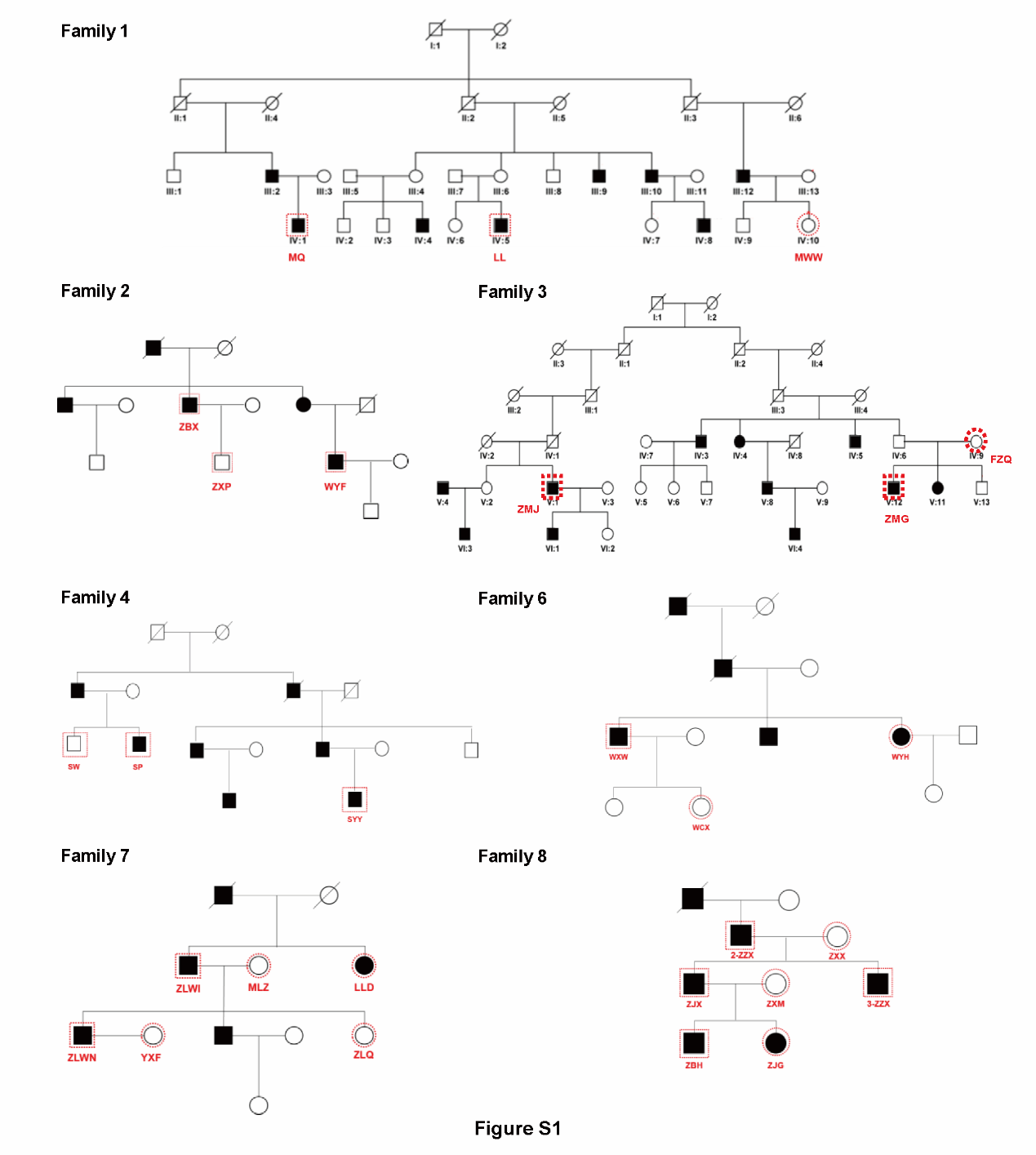

Supplement: Supplementary file 1 — Additional file 1: Figure S1. Samples from stuttering families (1-4 and 6-8) were selected for exome sequencing as indicated by red dashed lines. [file 41065_2021_211_MOESM1_ESM.docx]

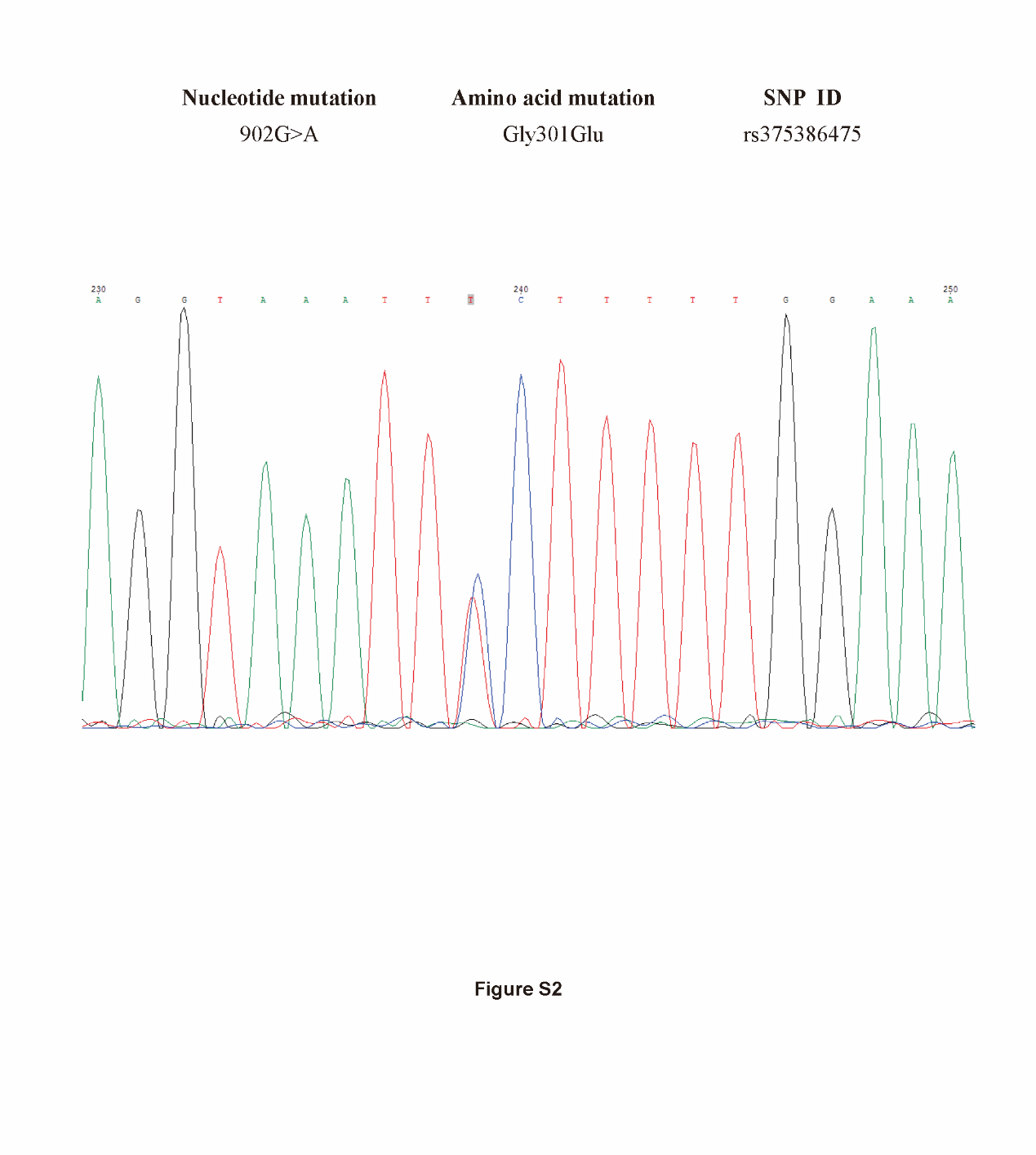

Supplement: Supplementary file 2 — Additional file 2: Figure S2. Confirmation of the presence of the IFNAR1 902G>A point mutation by Sanger sequencing. [file 41065_2021_211_MOESM2_ESM.docx]

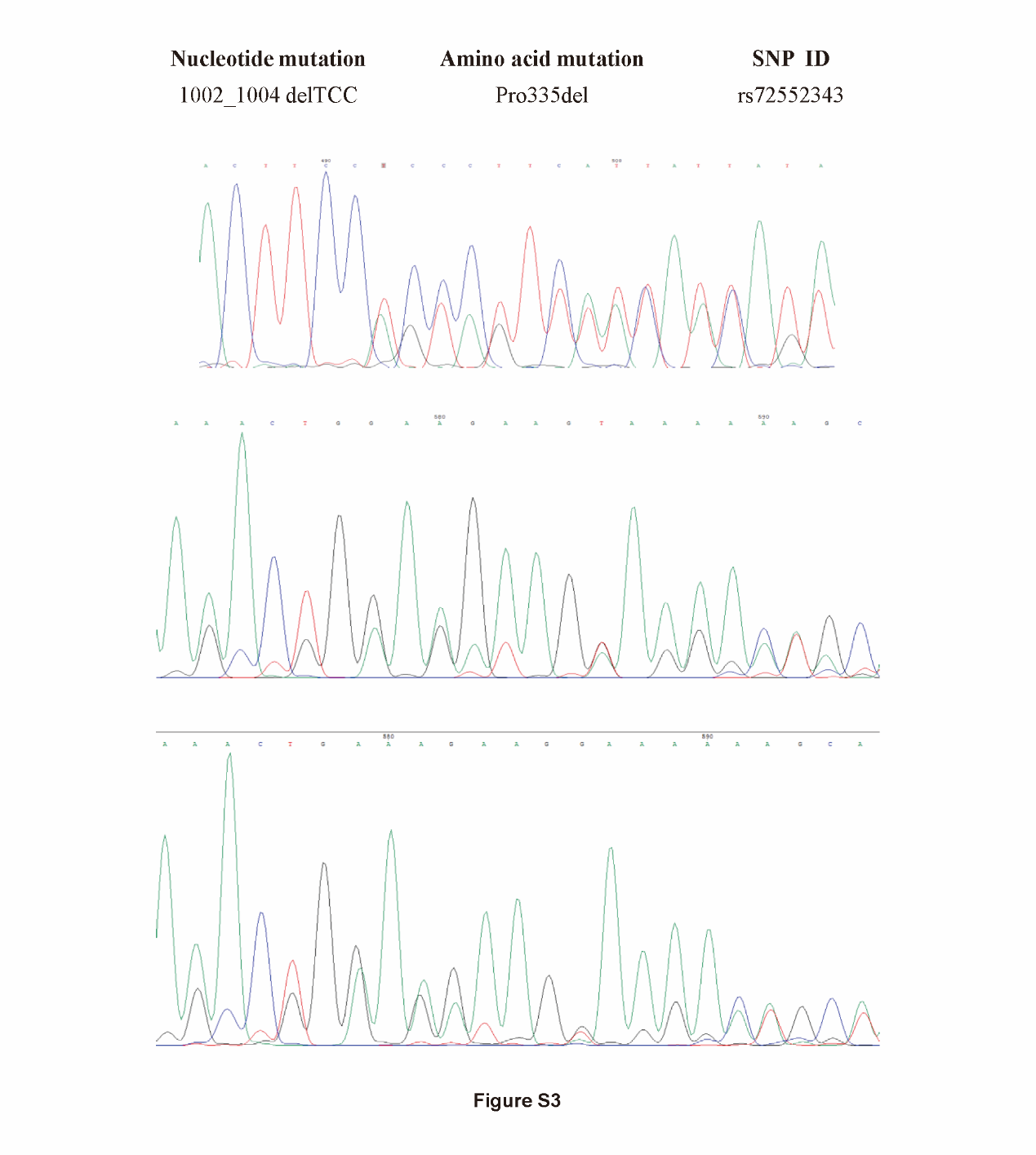

Supplement: Supplementary file 3 — Additional file 3: Figure S3. Confirmation of the presence of the IFNAR1 1002_1004 delTCC mutation by Sanger sequencing. [file 41065_2021_211_MOESM3_ESM.docx]
