# Supplementary material for: IFNAR1 gene mutation may contribute to developmental stuttering in the Chinese population
Source: Hereditas. 2021 Nov 18;158:46. doi: 10.1186/s41065-021-00211-y (PMC8600687; doi:10.1186/s41065-021-00211-y)
Supplement: Supplementary file 4 — Additional file 4: Supplementary Table S1. Samples evaluated in the present study. [file 41065_2021_211_MOESM4_ESM.docx]

Supplementary Table S1. Samples evaluated in the present study.
