# Supplementary material for: IFNAR1 gene mutation may contribute to developmental stuttering in the Chinese population
Source: Hereditas. 2021 Nov 18;158:46. doi: 10.1186/s41065-021-00211-y (PMC8600687; doi:10.1186/s41065-021-00211-y)
Supplement: Supplementary file 5 — Additional file 5: Supplementary Table S2. The WES analysis revealed 28 cosegregating SNVs corresponding to 24 candidate genes in Family 0. [file 41065_2021_211_MOESM5_ESM.docx]

Supplementary Table S2. The WES analysis revealed 28 cosegregating SNVs corresponding to 24 candidate genes in Family 0.
