# Supplementary material for: IFNAR1 gene mutation may contribute to developmental stuttering in the Chinese population
Source: Hereditas. 2021 Nov 18;158:46. doi: 10.1186/s41065-021-00211-y (PMC8600687; doi:10.1186/s41065-021-00211-y)
Supplement: Supplementary file 6 — Additional file 6: Supplementary Table S3. Functional annotation of IFNAR1 mutation using in silico prediction tools. [file 41065_2021_211_MOESM6_ESM.docx]

Supplementary Table S3. Functional annotation of IFNAR1 mutation using in silico prediction tools.

| **AA change** | **PolyPhen-2** | **SIFT** | **PROVEAN** | **PANTHER** |
| --- | --- | --- | --- | --- |
| Lys428Gln | Benign | Tolerated | Neutral | Probably benign |
| Leu552Pro | Possibly damaging | Deleterious | Deleterious | Probably benign |
| Gly301Glu | Probably damaging | Tolerated | Deleterious | Possibly damaging |
| Pro335del | N.A | N.A | Deleterious | N.A |
